# Adenovirus36 infection expresses cellular APMI and Visfatin genes in overweight Uygur individuals

**DOI:** 10.1186/1746-1596-9-83

**Published:** 2014-04-16

**Authors:** Yi Jiao, Xinmin Mao, Xi Chang, Kelimu Abudureyimu, Cheng Zhang, Jianfei Lu, Yanjiao Wang, Nuerbiye Nuermaimaiti, Yiliyasi Aisa, Xian Gong, Yaqun Guan

**Affiliations:** 1 Department of Biochemistry and Molecular Biology, Preclinical Medicine College, Xinjiang Medical University, Urumqi 830011, P. R China; 2 Traditional Chinese Medical College, Xinjiang Medical University, Urumqi 830011, P. R China; 3 Endoscopic Branch, Xinjiang Uygur Autonomous Regional People’s Hospital, Urumqi 830000, P. R China

**Keywords:** Adenovirus 36, Adipocytokines, Obesity, Adiponectin, Visfatin

## Abstract

**Objective:**

This study is to determine if Adenovirus type 36 (Ad36) infection is related to macrophage infiltration in the obese group and non-obese group and the related molecular mechanisms.

**Methods:**

Ninety obesity patients and 95 non-obesity Uygur individuals were enrolled in this study. CD68 levels in abdominal subcutaneous and omental adipose tissues were detected by immunohistochemistry. The cytokine expression levels of adiponectin (APMI) and visfatin in serum were measured by enzyme-linked immunosorbent assay. Infection of 3T3-L1 cells with Ad36 was performed. Real-time PCR was performed to determine expression levels of *APMI* and *Visfatin* genes in the 3T3-L1 preadipocytes infected with Ad36.

**Results:**

In the obese individuals infected with Ad36, the expression levels of adiponectin and visfatin in serum was elevated. For the individuals infected with Ad36, the macrophage infiltration (as indicated by CD68 level) in the obese group was also significantly higher than that in the non-obese group (P < 0.05) in both abdominal subcutaneous and omental adipose tissues. The real-time PCR results indicated that *APMI* mRNA levels and *Visfatin* mRNA levels in Ad36 infected cells were significantly increased.

**Conclusions:**

Ad36 infection may be a factor related with macrophage infiltration in adipose tissues of the obese patients. The *APMI* and *Visfatin* genes may be involved in the mechanism underlying the effect of Ad36 infection on the obese patients.

**Virtual Slides:**

The virtual slide(s) for this article can be found here: http://www.diagnosticpathology.diagnomx.eu/vs/1849614638119816

## Introduction

Obesity has become a severe public health problem in the world and affects people across various ethnicities in all countries [1]. Obesity is defined as excessive body fat accumulation that presents a risk to health. According to the Asia-Pacific adult obesity criteria, body mass index (BMI, a person’s weight divided by the square of his or her height) < 25 kg/m^2^ was considered as normal and BMI ≥ 25 kg/m^2^ was indicated as obese. Obesity increases the likelihoods of various diseases, especially heart disease, type 2 diabetes, sleep apnea, and some kinds of tumors [2-4]. Although obesity is usually resulted from a combination of excessive food intake and lack of physical exercises, it may be affected by factors involved in the metabolic processes, such as adiponectin and visfatin. Adiponectin, encoded by *APMI* gene, is a protein hormone that modulates metabolic processes, including glucose regulation and fatty acid oxidation [5]. It plays an important role in regulating body fat percentage, glucose level and lipid metabolism [6]. In adults, adiponectin level is inversely correlated with BMI, waist circumference (WC), body fat percentage and fasting plasma insulin concentration. In patients with obesity, type 2 diabetes or coronary artery disease, plasma adiponectin concentration is significantly reduced [7]. Visfatin is a protein secreted by visceral adipose tissue and has insulin-like metabolic effects on glucose metabolism [8]. Studies found that serum visfatin levels are increased in individuals with abdominal obesity and correlate strongly with the amount of visceral adipose tissue in humans [9,10]. The observed increase of visfatin in obesity may be a counterregulation preventing further glucose increase.

In addition, some infectious factors may be related to obesity [11,12]. It is reported that Adenovirus type 36 (Ad36) infection is associated with obesity. For example, Pasarica et al. [13] found that Ad-36 induced lipid accumulation in primary human adipose-derived stem/stromal cells (hASC). By using serum neutralization assay, Atkinson et al. [14] found that 30% of obese and 11% of non-obese humans have neutralizing antibodies to Ad36. These results indicate that Ad-36 plays a role in the etiology of human obesity. However, the underlying cellular and molecular mechanism of Ad36 in human obesity is still unclear. One of the Ad36 viral genes, *E4orf1*, is thought to be involved in the mechanism underlying virus-induced obesity by increasing adipocyte differentiation factors [15]. However, whether other factors regulating body fat such as adiponectin and visfatin are also involved in Ad36-induced obesity is unknown and needs further investigation.

We found that a big portion of the long-term obese Uighur residents in Xinjiang region, China were naturally infected with Adenovirus type 36 (Ad36). In this study, we aimed to determine if Ad36 infection was related to obesity in Uighur residents in Xinjiang region and the underlying mechanisms. Obese individuals and non-obese controls who were long-term Uighur residents in Xinjiang region, China were enrolled. Biochemical indices were compared between obese and non-obese individuals (with or without Ad-36 infection). The macrophage infiltration (as indicated by CD68 level) in abdominal subcutaneous and omental adipose tissues was analyzed. The level of adiponectin and visfatin in the serum and the mRNA expression of adiponectin and visfatin in 3T3-L1 preadipocytes infected with Ad36 were also investigated.

## Materials and methods

### Patients

Ninety obesity patients and 95 non-obesity Uygur individuals were enrolled in this study. According to the Asia-Pacific adult obesity criteria, body mass index (BMI) < 25 kg/m^2^ was considered as normal and BMI ≥ 25 kg/m^2^ was indicated as obese. The age range of the individuals was 30-70 years old. All individuals enrolled in this study were long-term Uighur residents in Xinjiang region, China. There were no blood relationships among them. Individuals with the following characteristics were excluded: (i) artery or peripheral vascular disease; (ii) thyroid dysfunction; (iii) acute inflammatory and trauma and other emergency situation, and (iv) malignancies and a history of alcohol or drugs. All studies were conducted under the hospital ethics committee approval and informed consent from every individual was obtained. The study was approved by the ethics review board of Xinjiang Uygur Autonomous Regional People’s Hospital.

### Specimen collection

To determine microphage infiltration in adipose tissue, biopsy of abdominal subcutaneous and omental adipose tissues was performed. Briefly, abdominal subcutaneous and omental adipose tissues were obtained by surgical biopsy from the periumbilical area, under local anesthesia (1% xylocaine). First, the skin was cleaned and covered with special surgical drapes. An incision of < 0.5 cm was made with a plain scalpel to access the subcutaneous and omental adipose tissues. About 500 mg adipose tissues was then removed, washed in sterile saline solution, placed immediately in liquid nitrogen, and stored at -80°C until analysis. The skin incision was then closed with absorbable suture material.

Venous blood (5 mL) was also collected from cubital vein. The serum isolated from venous blood was used for detection of biochemical indices.

### Analysis of biochemical indices

Cholesterol oxidase test with sensitivity of 0.08 mmol/L was performed to test the total cholesterol (TC). Polyethylene sulfate test with sensitivity of 0.078 mmol/L was performed to test low density lipoprotein cholesterol (LDL-C) levels. Enzymatic assay test was performed to determine the triglyceride (TG) (Sensitivity: 0.05 mmol/L) and high density lipoprotein cholesterol (HDL-C) (Sensitivity: 0.01 mmol/L). Glucose oxidase-peroxidase-coupled method with sensitivity of 0.11 mmol/L was used to measure the fasting blood glucose (FBG) levels.

### Enzyme-linked immunosorbent assay (ELISA)

The human serum cytokine concentrations of adiponectin and visfatin were measured by ELISA kit purchased from Beijing Aidi Bo biological products company (Beijing, China). The optical density of each well was determined by a microplate reader at a wave length of 450 nm. Standard curve was generated to calculate the concentration of each sample.

### Immunohistochemical staining

Adipose tissue was dehydrated and embedded to make paraffin section. Primary antibodies against CD68 (1:50) was used. Hematoxylin staining was performed. Five views were randomly selected under light microscope from each slice. Image-pro plus 6.0 software analysis was used to process the results. The total OD values indicating CD68 expression levels were calculated.

### Ad36 infection

The 3T3-L1 preadipocytes were infected with adenoviruses for 1 h with a titer of 3.8 MOI or 7.6 MOI or mock infected with medium as a control. Each infection was performed in triplicates. After infection, the cells were cultured in complete DMEM containing 10% fetal bovine serum and 1% penicillin-streptomycin (Sigma-Aldrich, USA) in an incubator with the condition of 37°C and 5% CO2. The complete medium was changed every two days. At day 1, 3, 5, and 7 days after infection the viruses were collected, respectively, for further analyses.

### Quantitative reverse transcription-PCR (RT-PCR)

The total RNA was isolated from Ad36 infected preadipocytes using Trizol (Invitrogen Corporation, USA). The RT-PCR experiments were repeated at least 3 times. RNA (1 μg) was reverse-transcribed into cDNA using Oligo (dT)15 in a reverse transcription II system (Promega, Madison, WI, USA), according to the manufacturer’s instructions. Expression of mRNAs was quantified by quantitative PCR using an ABI Prism Sequence Detection System (Applied Biosystems). Primers for *β*-*actin*, *APMI*, *Visfatin* and *E4orf1* genes were given in Table 1. Template-negative and RT-negative conditions were used as controls. Gene amplification was monitored using the ABI 7500 software. The corresponding amplification plots were used to determine the threshold cycle value. And the threshold cycle value was defined as the number of PCR cycles taken for fluorescent intensity to reach a fixed threshold for each signal. The relative amounts of *APMI*, *Visfatin* and *E4orf1* mRNA were normalized to the amount of *β-actin* mRNA in the same sample.

**Table 1:** Primers used in this study

**Gene name**	**Primers**	**Primer sequences**
*β*-*actin*	Forward	5’-CCACCATGTACCCAGGCATT-3’
	Backward	5’-AGGGTGTAAAACGCAGCTCA-3’
*APMI*	Forward	5’-GGGGACCACAATGGACTCTA-3’
	Backward	5’-GGTGTATGGGCTATGGGTAGTT-3’
*Visfatin*	Forward	5’-CGCACTACCTGGCTCAAGA-3’
	Backward	5’-CCTTTGTGAAGAGGAGGAGACT-3’
*E4orf1*	Forward	5’-GCATACTAACCCAGTCCGATG-3’
	Backward	5’-AATCACTCTCTCCAGCAGCAGG-3’

Note: APMI, adiponectin.

### Statistical analyses

The experimental data were given as mean ± standard error (SEM). Statistical software (SPSS15.0, Chicago, IL, USA) was used for independent sample *t* test, followed by one-way variance analysis. Levels of mRNA transcripts were analyzed with *t*-test by SPSS15.0 statistical software. P < 0.05 indicated a significant difference.

## Results

### Clinical characteristics, biochemical indices, and changes in cytokine expression levels in the obese and non-obese group

To investigate the differences in the obese and non-obese groups, the data of body mass index (BMI), waist circumference (WC), hip circumference (HC), diastolic blood pressure (DBP), systolic blood pressure (SBP), FBG, TC, TG, HDL-C, LDL-C, adiponectin, and visfatin between the two groups were compared (Table 2). Compared with non-obese group, obese group had significantly higher levels of BMI, WC, HC, DBP, TC, TG and visfatin (P < 0.05). However, the serum levels of adiponectin in the obese group were significantly lower than the levels in the non-obese group (P < 0.05).

**Table 2:** **Clinical characteristics and biochemical indices in the obese and non**-**obese groups** (**mean** ± **SEM**)

**Parameters**	**Non-obese group &**	**Obese group**^**#**^	**Non-obese group**		**Obese group**	
			**Ad36 (-) subgroup**	**Ad36 (+) subgroup**	**Ad36 (-) subgroup**	**Ad36 (+) subgroup**
Case number	95	90	57	38	37	53
Age	50 ± 10	53 ± 8	50 ± 10	49 ± 11	52 ± 8	54 ± 8
BMI (kg/m^2^)	22.4 ± 2.1	29.7 ± 3.7*	22.5 ± 2.3	22.3 ± 1.8	29.8 ± 3.9	29.6 ± 3.6
WC (cm)	84.6 ± 9.8	99.8 ± 8.4*	84.8 ± 10.5	84.5 ± 8.9	100.0 ± 8.2	99.5 ± 8.6
HC (cm)	95.5 ± 5.6	108.2 ± 8.0*	95.6 ± 4.9	95.3 ± 6.7	107.4 ± 8.2	108.9 ± 7.8
DBP (mmHg)	73.4 ± 10.6	80.9 ± 16.8*	73.5 ± 11.6	73.2 ± 9.1	81.2 ± 18.6	80.6 ± 15.3
SBP (mmHg)	118.2 ± 16.20	124.8 ± 21.2	119.2 ± 18.2	116.5 ± 12.8	125.2 ± 20.9	124.5 ± 21.7
FBG (mmol/L)	5.75 ± 2.09	5.82 ± 1.40	6.02 ± 2.40	5.35 ± 1.47	5.98 ± 1.71	6.04 ± 2.37
TC (mmol/L)	4.00 ± 0.84	4.34 ± 0.72*	3.95 ± 0.85	4.09 ± 0.85	4.37 ± 0.75	4.33 ± 0.69
TG (mmol/L)	1.42 ± 0.90	1.81 ± 0.88*	1.35 ± 0.96	1.51 ± 0.81	2.05 ± 1.15	1.59 ± 0.47**
HDL-C (mmol/L)	1.35 ± 0.60	1.28 ± 0.58	1.25 ± 0.37	1.50 ± 0.81	1.28 ± 0.58	1.29 ± 0.60
LDL-C (mmol/L)	2.28 ± 0.75	2.43 ± 0.65	2.23 ± 0.86	2.34 ± 0.56	2.34 ± 0.64	2.51 ± 0.65
Adiponectin (ng/ml)	0.59 ± 0.24	0.49 ± 0.15*	0.57 ± 0.24	0.62 ± 0.23	0.45 ± 0.13	0.54 ± 0.16**
Visfatin (ng/ml)	1.07 ± 0.39	1.23 ± 0.35*	0.98 ± 0.37	1.21 ± 0.40**	1.11 ± 0.35	1.32 ± 0.30**

Note: &, includes the Ad36 (-) non-obese subgroup and Ad36 (+) non-obese subgroup.

^#^, includes the Ad36 (-) obese subgroup and Ad36 (+) obese subgroup.

*, P < 0.05, when compared with the non-obese group.

**, P < 0.05, when compared with Ad36 (-) non-obese subgroup or Ad36 (-) obese subgroup, respectively.

*BMI*, body mass index. *WC*, waist circumference. *HC*, hip circumference. *DBP*, diastolic blood pressure. *SBP*, systolic blood pressure. *FBG*, fasting blood glucose. *TC*, total cholesterol. *TG*, triglyceride. *HDL*-*C*, high-density lipoprotein cholesterol. *LDL*-*C*, low-density lipoprotein cholesterol.

In non-obese group (n = 95), there were 57 individuals without Ad36 infection and 38 individuals with Ad36 infection. In obese group (n = 90), there were 37 patients without Ad36 infection and 53 patients with Ad36 infection. The difference in biochemical indices and in cytokine expression levels between obese patients with and without Ad36 was further analyzed. As shown in Table 2, in non-obese group, serum visfatin level in non-obese subgroup with Ad36-infection was significantly higher than that in non-obese subgroup without Ad36-infection (P < 0.05). In the obese group, the serum levels of TG, adiponectin and visfatin were significantly different between the obese individuals infected with Ad36 or not (P < 0.05). The TG levels in Ad36-infected obese subgroup were significantly decreased, while serum levels of adiponectin and visfatin in Ad36-infected obese subgroup were significantly increased. These results indicate that Ad36 infection induces elevated levels of adiponectin and visfatin and improved TG level in the obese individuals.

### The comparison of macrophage infiltration between the obese and non-obese group

In the subcutaneous adipose tissues of the obese and non-obese group, macrophage infiltration levels as indicated by the CD68 OD values (Table 3) in the obese group (14294.53 ± 2235.86) were higher than those in the non-obese group (10867.55 ± 2239.87). The difference was statistically significant (P < 0.05). For the individuals infected with Ad36, the macrophage infiltration in the obese group (14730.16 ± 2227.39) was also significantly higher than those (12258.06 ± 1025.58) in the non-obese group (P < 0.05).

**Table 3:** **Levels** (**OD values**) **of CD68 in subcutaneous adipose tissues and omental adipose tissues in the the obese and non**-**obese groups** (**mean** ± **SEM**)

**Tissues**	**Non-obese group &**	**Obese group**^**#**^	**Non-obese group**		**Obese group**	
			**Ad36 (-) subgroup**	**Ad36 (+) subgroup**	**Ad36 (-) subgroup**	**Ad36 (+) subgroup**
Subcutaneous adipose tissues	10867.55 ± 2239.87	14294.53± 2235.86*	10928.35 ± 1817.56	12258.06 ± 1025.58	10786.50 ± 2772.80	14730.16 ± 2227.39**
Omental adipose tissue	9316.35 ± 1172.26	12982.64 ± 1929.89	9332.27 ± 1096.35	12914.70 ± 2132.08	11723.17 ± 2888.25	12685.47 ± 1729.52**

Note: &, includes the Ad36 (-) non-obese subgroup and Ad36 (+) non-obese subgroup.

^#^, includes the Ad36 (-) obese subgroup and Ad36 (+) obese subgroup.

*, P < 0.05, when compared with the non-obese group.

**, P < 0.05, when compared with Ad36 (-) non-obese subgroup or Ad36 n-) obese subgroup, respectively.

**Figure 1:**
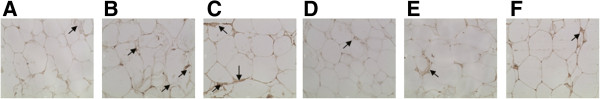
**CD68 expression in macrophages in the omental adipose tissues.** Adipose tissue was dehydrated and embedded to make paraffin section. Primary antibodies against CD68 (1:50) was used. Hematoxylin staining was performed (400 ×). Arrows indicated positive CD68-staining. **(A)** Ad36 (+); **(B)** Ad36(+), obese; **(C)** Ad36(+), non-obese; **(D)** Ad36 (-); **(E)** Ad36(-), obese; **(F)** Ad36(-), non-obese.

In the omental adipose tissues (Table 3, Figure 1) of the obese and non-obese group, the levels of macrophage infiltration in the obese group were significantly higher than those in the non-obese group, but the difference was not statistically significant (P > 0.05). For the individuals infected with Ad36, the macrophage infiltration levels in the obese group were significantly higher than those in the non-obese group, with the difference being statistically significant (P < 0.05). These results suggest that Ad36 infection may be a factor related with macrophage infiltration of the obese group and non-obese group.

### Infection with Ad36 increases expression of *APMI* and *Visfatin* genes in preadipocytes

To further investigate the relationship of Ad36 infection with obese, the effect of Ad36 infection on 3T3-L1 preadipocytes was determined. Adenoviruses with titers of 3.8 MOI or 7.6 MOI were used to infect the cells, respectively. The mock-infected cells were used as the negative controls. Real-time quantitative PCR was performed to detect mouse β-actin, adiponectin, visfatin and E4orf1 mRNA levels. β-actin served as an internal control.

**Figure 2:**
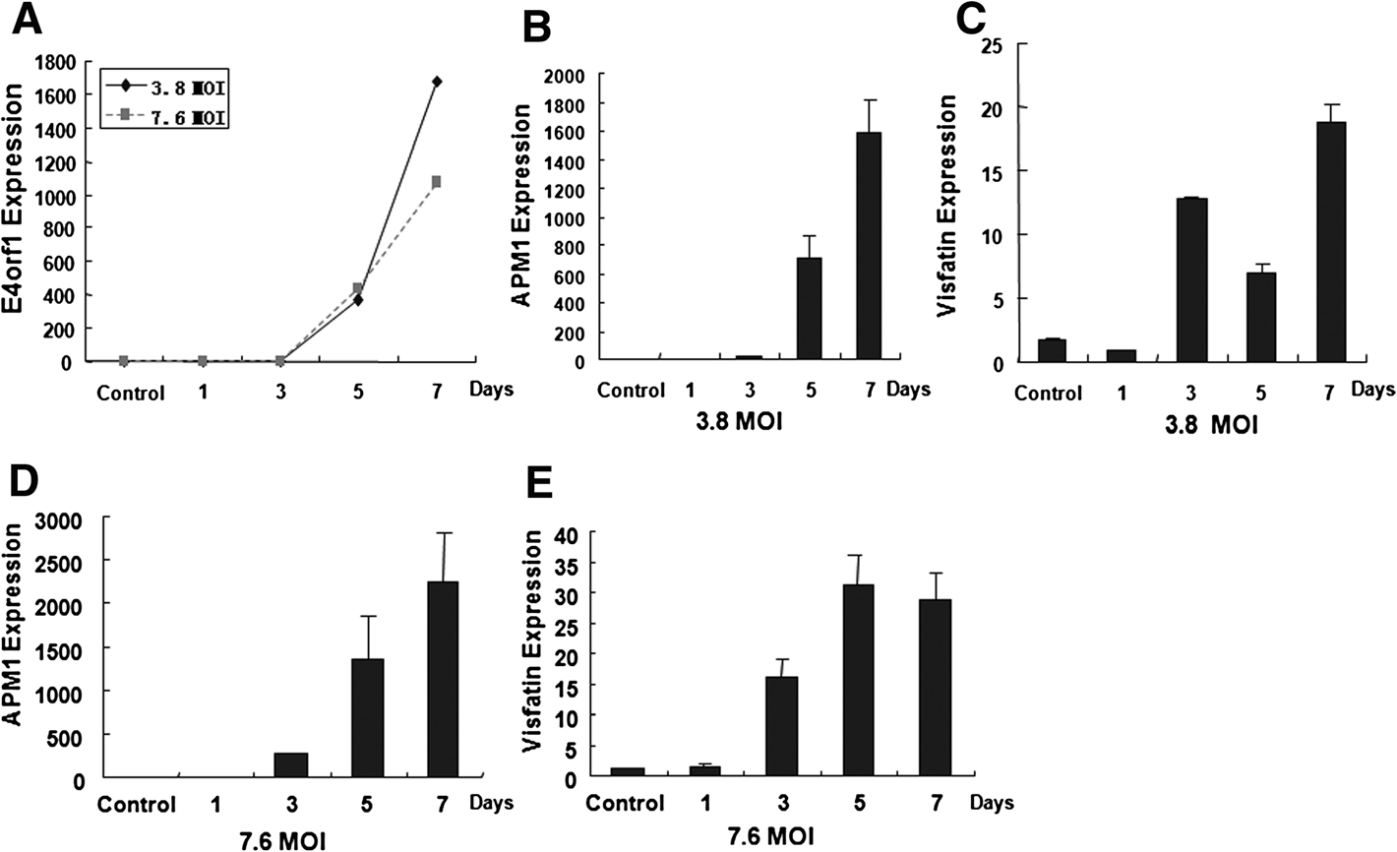
**Quantitative RT**-**PCR detection of *APMI *and *Visfatin *genes in preadipocytes infected with Ad36.** The 3T3-L1 preadipocytes were infected with Ad36 (3.8 MOI or 7.6 MOI). The mock-infected cells were used as the negative controls. The cells were harvested at day 1, 3, 5, 7 days after infection. The total RNAs were extracted and quantitative RT-PCR was performed to determine the mRNA levels. Adenoviruses with titers of 3.8 MOI or 7.6 MOI were used to infect the cells, respectively. **(A)***E4orf1* expression levels in cells infected with Ad36 (3.8 MOI or 7.6 MOI); **(B)***APMI* mRNA levels in cells infected with Ad36 of 3.8 MOI; **(C)***Visfatin* mRNA levels in cells infected with Ad36 of 3.8 MOI; **(D)***APMI* mRNA levels in cells infected with Ad36 of 7.6 MOI; **(E)***Visfatin* mRNA levels in cells infected with Ad36 of 7.6 MOI.

As shown in Figure 2, at days 1 to 7, expression of *E4orf1* gene was increased, indicating that the 3T3-L1 preadipocytes were infected with Ad36 (Figure 2A). Infection of 3T3-L1 cells with Ad36 (3.8 MOI) suggested that *APMI* mRNA levels and *Visfatin* mRNA levels were increased from the day 3 and day 4 post-infection, respectively (Figure 2B and 2C) (P < 0.05). Infection of 3T3-L1 cells with Ad36 (7.6 MOI) suggested that *APMI* mRNA levels and *Visfatin* mRNA levels were more significantly increased from the day 3 and day 4 post-infection, respectively (Figure 2D and 2E). These results show that infection of preadipocytes with Ad36 increases gene expression of *APMI* and *Visfatin* genes, suggesting that *APMI* and *Visfatin* genes may be involved in the mechanism underlying the effect of Ad36 infection on the obese patients.

## Discussion

Obesity is an important disease in some regions, such as the Xinjiang region, China. It directly and indirectly increases the likelihoods of various diseases [2-4,16,17]. Thus it is of great importance to identify the related factors of obesity and to investigate the underlying mechanisms. It is found that human Ad36 increases adiposity in several animal models, including rodents and non-human primates [18,19]. Since Ad36 is associated with human obesity, it is important to determine the relationship between its infection and obesity [20,21]. In this study, the relationship between Ad36 infection and obesity and the underlying mechanism of Ad36 in obesity was investigated.

Our results showed that, in the obese group, the serum levels of TG, adiponectin and visfatin were found to be significantly different between the obese individuals infected with Ad36 or not. The TG levels in Ad36-infected obese subgroup were decreased, while serum levels of adiponectin and visfatin in Ad36-infected obese subgroup were increased. The results suggest that in the obese individuals infected with Ad36, the expression of adiponectin in serum was elevated and thus TG levels were improved. Previous studies have shown that Ad-36 infection is associated with reductions in serum TC and TG both in adults and children [14,22]. Thus, our data in obese patients from Xinjiang region were consistent with previous reports. And, our data further confirmed that Ad36 was related with obesity, suggesting that Ad36 might be involved in the etiology of obesity.

Studies have shown that inflammation contributes to the maintenance of the obesity state and that Ad36 may maintain the obesity state by inducing inflammation [23-25]. Adipose tissue in obese subjects is characterized by macrophage infiltration [26]. Thus, to determine whether Ad36 induces macrophage infiltration in adipose tissue, we detected the expression of CD68 in both abdominal subcutaneous and omental adipose tissues by immunohistochemistry. The expression of CD68 is indicative of macrophage infiltration. The results showed that, in the individuals with Ad36 infection, the macrophage infiltration in the obese group was also significantly higher than that in the non-obese group in both abdominal subcutaneous and omental adipose tissues. Our results, which were consistent with previous reports, indicate that macrophage infiltration may be involved in Ad36 induced obesity.

Ad36 infection accelerates the differentiation of preadipocytes to adipocytes in 3T3-L1 cells and human preadipocytes [13,27]. Although Ad36 *E4orf1* gene is thought to be involved in the mechanism underlying virus-induced obesity, other related molecular factors are undetermined. As previously reported [5-10], adiponectin and visfatin are two important factors involved in obesity. Our results showed that serum levels of adiponectin and visfatin were significantly increased in obese patients infected with Ad36. Thus, to investigate whether adiponectin and visfatin are involved in Ad36-induced obesity, real-time PCR was performed to determine expression levels of *APMI* and *Visfatin* genes in the 3T3-L1 preadipocytes infected with Ad36. The expression of Ad-36 *E4orf1* gene was increased, indicating that the 3T3-L1 preadipocytes were infected with Ad36. Infection of 3T3-L1 cells with Ad36 suggested that *APMI* mRNA levels and *Visfatin* mRNA levels were increased from the day 3 and day 4 post-infection, respectively. These results indicate that infection of preadipocytes with Ad36 increases gene expression of *APMI* and *Visfatin* genes, and further suggest that *APMI* and *Visfatin* genes may be involved in the mechanism underlying the effect of Ad36 infection on the obese patients. The mechanisms underlying the effect of Ad36 infection will be further investigated in the future.

In summary, our data showed that Ad36 was associated with obesity of patients from Xinjiang region. And, Ad36 infection may be a factor related with macrophage infiltration in adipose tissues of the obese patients. In addition, increased levels of adiponectin and visfatin might be the mechanisms underlying the effect of Ad36 infection on obesity.

## Competing interest

The author’s declare that they have no competing interests.

## Authors’ contributions

JY and GYQ collected the specimens, carried out the biochemical studies, participated in the immunohistochemistry assay and drafted the manuscript. MXM, CX and AK carried out the quantitative RT-PCR assay. ZC, LJF and WYJ performed the ELISA assay. NN, AY and GX did the Ad36 infection experiment. JY participated in the design of the study and performed the statistical analysis. GYQ conceived of the study, and participated in its design and coordination and helped to draft the manuscript. All authors read and approved the final manuscript.
